# Evaluating the Types of Performance Monitoring Used in Private Chartered Universities in Uganda: A Mixed-Methods

**DOI:** 10.12688/f1000research.166395.3

**Published:** 2025-10-23

**Authors:** Turyamureeba silaji, Zulaihatu Lawal Bagiwa, Tukur Muhammad

**Affiliations:** 1 Educational-foundations, Kampala International University - Western Campus, Bushenyi, Western Region, Uganda; 2 Educational-foundations, Kampala International University - Western Campus, Bushenyi, Western Region, Uganda; 3 Science- Education, Kampala International University - Western Campus, Bushenyi, Western Region, Uganda

**Keywords:** Types of Performance Monitoring, Evaluating, Private Chartered Universities, A mixed-methods, Uganda

## Abstract

**Background:**

Performance monitoring is essential for enhancing academic staff productivity and institutional accountability in higher education. In Uganda, private chartered universities face challenges in implementing effective performance monitoring systems. This study examines the types and effectiveness of these systems in private chartered universities in Western Uganda.

**Methods:**

The study was guided by Fayol’s Administrative Management Theory and Vroom’s Expectancy Theory. A concurrent triangulation mixed-methods design was employed. Quantitative data were collected from 386 academic staff using structured questionnaires, while qualitative data were gathered through in-depth interviews with 10 academic deans from four selected universities. Ethical approval was obtained from institutional review boards and the Uganda National Council for Science and Technology (SS3145ES). Descriptive statistics, correlation, and regression analyses were used to analyze quantitative data, while thematic analysis was used for qualitative responses.

**Results:**

Findings indicate that various performance monitoring tools are in use, including student evaluations, peer reviews, and supervisory assessments. However, their application is often inconsistent, with limited feedback loops and weak integration into staff development initiatives. Quantitative analysis revealed a moderate positive correlation between effective performance monitoring and academic staff performance (r = 0.48, p < 0.05). Regression analysis showed that performance monitoring accounted for 21.3% of the variance in staff performance.

**Conclusions:**

While performance monitoring tools are present in private chartered universities in Western Uganda, their inconsistent implementation undermines their effectiveness. Strengthening the integration of monitoring outcomes into professional development and decision-making processes could enhance academic staff performance. These findings underscore the need for systematic policy enforcement and capacity-building in performance management practices within the sector.

## Introduction

Effective performance monitoring is essential for achieving organizational goals in higher education institutions. It involves the systematic evaluation of academic staff’s contributions to teaching, research, and service, ensuring that institutional objectives are met. In private universities, where competitiveness and accountability are heightened, performance monitoring becomes even more critical. In Uganda, the higher education sector has witnessed significant growth, with private chartered universities playing a pivotal role in expanding access to tertiary education. However, this expansion has brought challenges related to maintaining academic standards and staff performance. Previous studies have indicated that performance monitoring practices in private universities are often coercive and unsustainable, failing to enhance quality teaching and research effectively. This study explores how performance monitoring is practiced in private chartered universities in Western Uganda and its implications for academic staff performance. By examining the types of performance monitoring systems in place and their effectiveness, the research aims to provide insights into how these practices influence academic staff performance and suggest ways to enhance their efficacy.

## Methodology

### Research design

This study adopted a **cross-sectional and correlational design**, integrated within a **concurrent triangulation mixed-methods framework**. The cross-sectional approach enabled data collection at a single point in time from academic staff and deans, while the correlational design tested the strength and direction of relationships between performance monitoring and academic staff performance. Integration occurred at the interpretation phase, where qualitative insights were used to explain statistical relationships.

This study employed a concurrent triangulation mixed-methods design, wherein both quantitative and qualitative data were collected simultaneously to provide a comprehensive understanding of performance monitoring practices in private chartered universities in Western Uganda. This design facilitates the corroboration of findings across different data sources, enhancing the validity of the results. The study was guided by Fayol’s Administrative Management Theory and Vroom’s Expectancy Theory. A concurrent triangulation mixed-methods design was employed. Quantitative data were collected from 386 academic staff using structured questionnaires, while qualitative data were obtained through in-depth interviews with 10 academic deans from four selected universities. Ethical approval was obtained from the relevant institutional ethics review boards and the Uganda National Council for Science and Technology (Ref: SS3145ES). Informed consent was obtained from all participants prior to data collection. For the questionnaire respondents, written informed consent was secured electronically through a consent form attached to the survey. For the in-depth interviews, verbal informed consent was obtained and recorded with participant approval, as some participants preferred not to sign documents due to confidentiality concerns. This approach was reviewed and approved by the ethics committees.

Quantitative data were gathered through structured questionnaires administered to academic staff, while qualitative insights were obtained via in-depth interviews with academic deans. The integration of these datasets occurred during the interpretation phase, allowing for a nuanced analysis that captures both statistical trends and contextual factors influencing performance monitoring systems. Mixed-methods studies provide valuable insights into staff experiences and perceptions of institutional performance practices ( Ezzeddine et al., 2023). Applying such approaches ensures that both qualitative experiences and quantitative outcomes inform policy adaptations.

### Study location

The research was conducted across two private chartered universities located in the Western region of Uganda:

These institutions were selected based on their accreditation status and representativeness of private higher education in the region.

### Participant recruitment and selection

Participants included academic staff and academic deans from the aforementioned universities.

• Academic Staff: A total of 386 academic staff members were selected using stratified random sampling to ensure representation across faculties and departments. Inclusion criteria encompassed full-time employment status and a minimum of one year of teaching experience at the current institution. Exclusion criteria included part-time lecturers and administrative staff without teaching responsibilities.
• Academic Deans: Ten academic deans were purposively selected for in-depth interviews, based on their leadership roles and direct involvement in performance monitoring processes within their respective faculties.

Recruitment occurred between January and March 2025, with invitations sent via institutional email and follow-up meetings conducted to obtain informed consent.

### Data collection instruments

The Cronbach’s Alpha coefficients for the main constructs were as follows: performance monitoring (α = 0.84), staff performance (α = 0.87), and organizational structure (α = 0.82), confirming internal consistency. For qualitative data, a preliminary coding framework was developed with three main themes: (1) monitoring mechanisms, (2) feedback and developmental use, and (3) challenges and perceptions.

Data were collected using structured questionnaires and interview guides. The questionnaires were administered to the 386 academic staff to generate quantitative data, while interviews with the 10 Deans provided qualitative insights. To ensure validity, the Content Validity Index (CVI) was computed after expert review of the questionnaire, and the interview guides were validated through expert consultation to ensure clarity, relevance, and content adequacy. Reliability of the questionnaire was established through Cronbach’s Alpha coefficient, which confirmed internal consistency, while reliability of the interview guide was enhanced through pretesting with a small group of academic leaders who were not part of the main study.

### Data analysis

Quantitative data were coded and entered into the Statistical Package for Social Sciences (SPSS) for analysis. Descriptive statistics such as frequencies, percentages, and standard deviations were used to summarize the data, while inferential statistics, including correlation analysis, ANOVA, and regression, were employed to test relationships between organizational structures, performance monitoring, and academic staff performance. Qualitative data obtained from the Deans were transcribed, categorized, and thematically analyzed using NVivo 12 software, which facilitated coding, retrieval, and organization of emerging themes.

Before conducting regression analysis, assumptions of normality, linearity, and multicollinearity were tested and met (VIF < 2.0; tolerance > 0.5). The R ^2^ value of 0.213 implies that approximately 21.3% of the variance in academic staff performance is explained by performance monitoring effectiveness. While moderate, this effect is practically meaningful, suggesting that targeted improvements in feedback and developmental use could yield substantial performance gains.

### Data integration

Integration of findings occurred at both the analysis and interpretation stages. Quantitative results provided the magnitude and direction of relationships among variables, while qualitative findings enriched these results by explaining how and why certain patterns occurred. The joint interpretation of both strands provided a comprehensive and contextualized understanding of the influence of organizational structures and performance monitoring on academic staff performance in private chartered universities in Uganda.

### Demographic characteristics

The demographic profile of the academic staff participants was as follows:

• Gender: Approximately 60% male and 40% female.
• Age Distribution: Majority (50.5%) aged between 30–39 years; 29.2% aged 40 and above; 20.3% aged below 30 years.
• Educational Qualifications: 61.0% held Master’s degrees; 23.3% held PhDs; 11.0% held Bachelor’s degrees; 4.7% held postgraduate diplomas.
• Academic Positions: Positions ranged from Teaching Assistants to Senior Lecturers, with a majority serving as Lecturers.

These demographics provide context for interpreting the findings and assessing the applicability of the results to similar institutional settings.

### Data analysis and interpretation

Quantitative Data: Data from the structured questionnaires were analyzed using the Statistical Package for the Social Sciences (SPSS) version 26. Analytical procedures included:

• Descriptive Statistics: To summarize demographic information and key variables.
• Pearson Correlation Coefficient: To assess the relationship between performance monitoring effectiveness and academic staff performance (r = 0.48, p < 0.05).
• Linear Regression Analysis: To determine the extent to which performance monitoring predicts academic staff performance (R ^2^ = 0.213, F(1, 384) = 49.79, p < 0.001).

No significant missing data were reported; however, any incomplete responses were excluded from specific analyses to maintain data integrity.

Qualitative Data: Interview transcripts were analyzed using thematic analysis, following Braun and Clarke’s six-phase framework. The process involved:

1. Familiarization with the data
2. Generating initial codes
3. Searching for themes
4. Reviewing themes
5. Defining and naming themes
6. Producing the report

NVivo 12 software facilitated the coding and organization of qualitative data. Emergent themes included perceptions of performance monitoring tools, challenges in implementation, and suggestions for enhancing effectiveness.

The integration of quantitative and qualitative findings provided a comprehensive understanding of performance monitoring practices, highlighting both measurable outcomes and contextual nuances.

### Literature review

Performance monitoring is a cornerstone of human resource management and organizational development in higher education. According to Armstrong (2020), performance monitoring enhances productivity when feedback mechanisms are timely, actionable, and aligned with strategic objectives. Ololube (2015) highlights that evaluation systems must be fair, transparent, and linked to both organizational and individual development. Previous studies have shown that performance monitoring practices in private universities are often coercive and bureaucratic, limiting their effectiveness in enhancing teaching and research quality ( Atwebembeire & Malunda, 2019).

Recent evidence also indicates that staff accreditation and competency frameworks can enhance overall university performance, particularly when aligned with institutional goals ( Albaroudi & Alenezi, 2025). This supports the need for context-sensitive performance monitoring mechanisms recommended in our study.

In the Ugandan context, Okello and Kasozi (2021) report significant disparities in the application of performance monitoring across private universities, often resulting in staff dissatisfaction and disengagement. Nyeko and Tumwine (2022) assert that performance monitoring has little effect on academic output unless followed by actionable feedback and institutional support.

Studies have shown that Ugandan universities often rely on mechanistic performance monitoring systems that emphasize bureaucracy, which may negatively affect academic staff performance ( Kato & Tumwine, 2020; Nsubuga & Nalukwago, 2021). In line with these observations, a study on practices and challenges in private universities in Uganda explored the performance monitoring mechanisms employed and their effectiveness. Anchored in Performance Management Theory, the study focused on both academic and administrative personnel and adopted a descriptive survey design with a quantitative approach. Data were analyzed using descriptive statistics and correlation analysis. The findings indicated that private universities primarily use performance appraisals, peer reviews, and student evaluations as monitoring tools, contributing to a 15% improvement in staff performance and a 10% increase in institutional effectiveness. However, challenges such as inconsistent implementation and inadequate feedback mechanisms were identified. The study therefore recommended standardizing monitoring practices, providing regular training for evaluators, and enhancing feedback systems to improve effectiveness. These results reinforce the importance of structured performance monitoring in improving institutional outcomes, consistent with findings from similar studies.

In addition to the above study, they conducted another research titled “Performance Monitoring Mechanisms in Private Universities in Uganda: Practices and Challenges.” The study aimed to explore the various performance monitoring mechanisms utilized by private universities in Uganda and evaluate their effectiveness. The authors hypothesized that these mechanisms have a significant influence on staff performance and institutional efficiency. Anchored in Performance Management Theory, the study concentrated on both academic and administrative personnel in private universities. A descriptive survey design with a quantitative approach was employed, with data analyzed through descriptive statistics and correlation analysis. The findings revealed that private universities primarily use performance appraisals, peer reviews, and student evaluations as performance monitoring tools, resulting in a 15% improvement in staff performance and a 10% increase in institutional effectiveness. However, challenges such as inconsistent implementation and lack of feedback mechanisms were noted. The study recommended standardizing performance monitoring practices, providing regular training for evaluators, and enhancing feedback mechanisms to improve effectiveness. These findings support the importance of structured performance monitoring as highlighted in similar studies.

In a similar study, Nsubuga and Nalukwago (2021) investigated how performance monitoring systems influence faculty productivity and job satisfaction in private higher education institutions in Uganda. Their study aimed to assess whether effective performance monitoring systems enhance faculty productivity and job satisfaction, guided by Goal Setting Theory. They conducted a cross-sectional survey focusing on faculty members, employing a quantitative approach with regression and factor analysis for data evaluation. The results showed that institutions with well-implemented performance monitoring systems experienced a 20% increase in faculty productivity and a 12% improvement in job satisfaction. Challenges identified included the need for clearer performance metrics and better integration with faculty development programs. The study recommended developing clear performance metrics, integrating monitoring systems with professional development, and ensuring transparent evaluation processes. This research aligns with the findings on the effectiveness of structured performance monitoring in enhancing productivity and satisfaction.

While, Waiswa and Kiggundu (2022) investigated the challenges and best practices in monitoring academic staff performance in private universities in Uganda in their study, “Challenges and Best Practices in Performance Monitoring for Academic Staff in Private Universities in Uganda.” They sought to identify the challenges and best practices associated with performance monitoring to improve staff performance and institutional outcomes, using Performance Improvement Theory. The qualitative case study employed mixed methods, including thematic and comparative analysis. The study found that common challenges included a lack of uniform monitoring standards and inadequate feedback, while best practices identified were regular performance reviews, clear goal setting, and staff involvement in developing performance metrics. These practices led to improved performance and higher staff morale. The study recommended adopting uniform performance monitoring standards, involving staff in metric development, and providing regular, constructive feedback. This research builds on existing knowledge by emphasizing the benefits of structured and inclusive performance monitoring practices.

However, Kato and Tumwine (2020) conducted a study titled Performance Monitoring Mechanisms in Private Universities in Uganda: Practices and Challenges, aiming to investigate how different performance monitoring mechanisms impact staff performance and institutional effectiveness. Guided by Performance Management Theory, the study focused on academic and administrative staff, employing a descriptive survey design with a quantitative approach. The data were evaluated through the use of descriptive statistics and correlation analysis. The findings revealed that performance appraisals, peer reviews, and student evaluations significantly improved staff performance by 15% and institutional effectiveness by 10%. Despite these improvements, challenges such as inconsistent implementation and inadequate feedback mechanisms were noted. Consequently, the study recommended standardizing performance monitoring practices and enhancing feedback systems to improve overall effectiveness. These findings reinforce the importance of structured performance monitoring, aligning with similar research that emphasizes its impact on staff performance and institutional success.

In addition, Nsubuga and Nalukwago (2021) investigated the influence of performance monitoring systems on faculty productivity and job satisfaction in private higher education institutions in Uganda through their study titled “Assessing the Impact of Performance Monitoring Systems on Faculty Productivity in Private Higher Education Institutions in Uganda.” Their objectives were to determine how effective performance monitoring systems influence faculty productivity and job satisfaction, guided by Goal Setting Theory. Employing a cross-sectional survey design and a quantitative approach, the researchers engaged faculty members from various private universities and analyzed the data using regression and factor analysis. The study revealed that well-implemented performance monitoring systems resulted in a 20% boost in faculty productivity and a 12% increase in job satisfaction. However, challenges such as the need for clearer performance metrics and better integration with development programs were identified. The study recommended developing explicit performance metrics and integrating monitoring with professional development, further supporting the notion that structured performance monitoring enhances productivity and satisfaction.

Furthermore, Mugisha and Okello (2021) conducted a study to examine the role of performance monitoring in enhancing academic staff productivity in private universities in Uganda. Guided by the Theory of Performance Appraisal, their study utilized a mixed methods design involving surveys and interviews with academic staff and university administrators. Data analysis included quantitative statistical methods and qualitative thematic analysis. The results indicated that performance monitoring practices, such as regular appraisals and feedback, were associated with a 17% increase in staff productivity. Challenges included the need for more transparent appraisal processes and better alignment of performance metrics with institutional goals. The study recommended improving the transparency of appraisal processes and aligning performance metrics with institutional objectives, thus supporting existing literature on the importance of effective performance monitoring.

Whereas, Kagina and Mwesigwa (2022) carried out a study that centered on performance monitoring systems and their effect on the retention of academic staff in private universities. Using a quantitative approach with a survey design, they targeted academic staff across several private universities. Data analysis employed regression and correlation techniques. The study found that robust performance monitoring systems were linked to a 25% reduction in staff turnover and a 15% increase in job satisfaction. Notable challenges included inadequate support for performance improvement and lack of clear performance indicators. The study recommended strengthening support mechanisms and ensuring clear and consistent performance indicators. These findings contribute to the understanding of how performance monitoring systems can influence staff retention and satisfaction, aligning with previous research on the effectiveness of performance monitoring.

While, Amoah and Minishi-Majanja (2022) conducted a study to review the key performance indicators (KPIs) in university libraries in Ghana. The objective was to identify the KPIs utilized by these libraries and assess their effectiveness in managing staff performance. The findings showed that, while most staff members were unfamiliar with the specific KPIs in their libraries, they understood the concept and acknowledged its importance in measuring success and monitoring performance. The study also revealed that the majority of libraries lacked clear and measurable KPIs, with almost 80% of them not having explicitly defined KPIs known to staff. It was found that KPIs varied between libraries, often influenced by the unique mission and vision statements of each institution. To enhance staff performance in Ghanaian university libraries, the study recommends that management develop clear and measurable outcome-based metrics. These metrics should not only meet client needs but also improve the efficiency of information provision. Additionally, management should ensure that these KPIs are well-defined and communicated to library staff.

In a similar vein, Lamantinulu (2016) conducted research to identify and prioritize key performance indicators (KPIs) associated with teaching, learning, and the academic environment in private higher education institutions in Sulawesi Selatan, Indonesia. The process of prioritizing KPIs included identifying, validating, specifying, and analyzing the weight of each factor. The study used the average value determination method for important factors in KPI formulation and the Analytical Hierarchy Process (AHP). The study established 22 KPI formulations for teaching and learning aspects and nine for the academic atmosphere. AHP analysis revealed eight priority levels for teaching and learning KPIs and five for academic atmosphere KPIs. The KPI with the highest priority for teaching and learning was the percentage of the syllabus available to students and lecturers (ITE.3), assigned a weight of 0.13. For the academic atmosphere, the most important KPI was the level of interaction through activities like seminars, symposia, and workshops (IAA.2), with a weight of 0.197. The originality of this study is highlighted in its detailed formulation of these factors into KPIs that are crucial for study programs in private higher education, emphasizing the significance of prioritized KPIs on teaching, learning, and the academic environment.

In another study Bawack and Kamdjoug (2020) investigated how technology is used to improve learning in universities and other higher education institutions (HEIs), highlighting its ability to overcome the time and space limitations typical of traditional learning settings. Current studies primarily focus on the technology itself rather than the information it provides. Additionally, there is a dearth of research on technology adoption in universities and HEIs in developing countries, particularly in Africa. To address this gap, the authors proposed a model to explain how students’ information behaviors are evolving in the digital age and their impact on learning outcomes. They collected data from 303 students using a questionnaire and analyzed it with structural equation modeling partial least squares (SEM-PLS). The findings revealed that the model accounts for 60.2% of student satisfaction, 24.2% of academic performance, 24.1% of information sharing, and 19.8% of information exchange behavior. The study confirms that the use of digital information and its influencing factors significantly affect students’ experiences in college. These results are important for advancing information systems research and practice, especially in the development and assessment of educational technology.

Similarly, Ishaq, Azan, Rosdi, Abid, and Ijaz (2020) explored the growing significance and widespread use of information and communication technology (ICT) in education for students. Their research aimed to assess the various impacts of ICT on higher education. Specifically, the study investigated the relationship between ICT usage and academic performance among students in both public and private institutions in Pakistan. The study had several objectives: to understand why students access ICT services, to explore the frequency and duration of ICT use, and to describe the link between ICT use and academic performance. The research involved 300 students who completed a questionnaire. Data was analyzed using Pearson correlation coefficient and descriptive statistics to identify any correlation between ICT use and academic achievement. The findings revealed that most students had access to laptops or personal computers and the Internet at their universities. Many students reported using ICT to enhance essential skills and engage more effectively in their learning. The study concluded that the effective use of ICT has a significant positive impact on students’ academic performance.

Whereas, Alam, Ahmad, Naveed, Patel, Abohashrh, and Khaleel (2021) conducted a study highlighting that E-Learning has emerged as the main alternative to traditional face-to-face education during the global COVID-19 lockdown. Academic institutions worldwide have significantly invested in E-Learning, transitioning many traditional courses to this format. Ensuring the success of E-Learning initiatives is essential for establishing it as a sustainable method of learning. The study aimed to propose a comprehensive E-Learning service framework to guarantee effective delivery and use, thereby supporting sustainable learning and improving academic performance. The researchers developed and empirically tested a theoretical model based on an extensive literature review. This model identifies a range of success factors and connects them to various success measures, such as learning outcomes and academic performance. Validated with data from 397 participants involved with E-Learning systems at the top five public universities in southern Saudi Arabia, using Partial Least Squares regression with Smart PLS software, the study identified five key factors Learner’s Quality, Instructor’s Quality, Information Quality, System Quality, and Institutional Quality as determinants of E-Learning service performance. These factors collectively account for 48.7% of the variance in the perceived usefulness of E-Learning services, 71.2% of the variance in E-Learning system usage, and 70.6% of the variance in students’ learning and academic performance. Thus, the framework supports the successful and sustainable adoption of E-Learning services.

Similarly, Basri, Alandejani, and Almadani (2018) conducted a study to explore the adoption of information communication technology (ICT) in universities and its effect on students’ academic performance. The research also investigated how factors such as gender, GPA, and student majors influenced the relationship between ICT use and academic success. Using a quantitative approach with a sample of 1,000 students from four Saudi universities, data on ICT adoption and student performance were collected. Structural equation modeling was used to validate the research model, with path analysis performed using the Analysis of Moment Structures (AMOS). The study found a relationship between ICT adoption and academic performance in a conservative setting. It also noted that ICT adoption had a greater positive impact on female students compared to male students, while students’ IT major did not significantly affect academic achievement. The study includes a discussion of the findings, limitations, and recommendations for future research, as well as implications for existing knowledge.

AT and Wekesa and Makhamara (2020) conducted a study to assess the impact of performance appraisal on employee performance at Kibabii University in Bungoma County, Kenya. The research focused on evaluating the effects of different appraisal methods, including evaluation, management by objectives (MBO), performance appraisal design, and the 360-degree appraisal method on employee performance. The study was based on Organizational Justice Theory, Goal Setting Theory, and Expectancy Theory. A descriptive research design was used, targeting 400 employees from various job groups at Kibabii University. Stratified and systematic random sampling methods were employed to select a sample of 200 respondents. Primary data was gathered using a structured questionnaire, which was validated through expert review and a pilot study with ten randomly chosen employees. Reliability was assessed using internal consistency techniques, resulting in a Cronbach Alpha Coefficient of 0.7.

Descriptive statistics, including mean, standard deviation, and percentages, were used for analysis, while inferential statistics involved bivariate correlation and multiple regression analysis to confirm the relationships between variables. The findings showed that evaluation positively and significantly affected employee performance (β = 0.218; P-Value < 0.05), MBO also had a positive and significant impact (β = 0.164; P-Value < 0.05), while performance appraisal design had a positive but non-significant effect (β = 0.011; P-Value > 0.05), and the 360-degree appraisal method significantly influenced employee performance (β = 0.172; P-Value < 0.05). The study concluded that various evaluation methods, including supervisor, peer, self, subordinate, customer, and trainer evaluations, significantly enhance employee performance. Additionally, improving MBO practices such as setting clear objectives, monitoring progress, rewarding achievements, and involving stakeholders was found to significantly boost performance. Enhancements in performance appraisal design, including fairness, goal orientation, and regular evaluations, also positively affected employee performance. Finally, better implementation of the 360-degree appraisal method, including comprehensive feedback and skill assessment, was shown to significantly improve employee performance.

Rashidi, Zhang and Li (2022) found that African countries have been adopting performance management tools developed in the West, which have not provided value to institutions in developing regions. This “copy and paste” approach has led to the implementation of outdated Performance Management Systems (PMS) that fail to ensure staff accountability for performance. Their paper reviews literature on PMS in higher education institutions (HEIs) as part of a project aimed at creating a customized PMS for quality assurance and enhancement in HEIs. The literature was gathered through a systematic review on the EBSCOhost database, using key terms and backward snowballing. The findings suggest that performance management in higher education can be improved by adapting existing systems to current conditions and enhancing them with information and communication technology tools. On the other hand, Motshegwa (2024) reported that globally, companies and governments have experimented with various performance management systems, which have evolved over time with the emergence of new systems. The article reviews performance management within the human resource management literature, tracing its history and focusing on the 360-degree performance management system. It discusses performance management issues in organizational settings, particularly the application of the 360-degree system. Although managing performance is crucial, the 360-degree evaluation system has both strengths and weaknesses that management should be aware of and address. The literature review highlights that while performance management is a valuable tool for measuring performance, the 360-degree system can be misused for personal vendettas or collusion. Despite these potential issues, adopting the 360-degree performance management system can improve organizational and employee performance by incorporating feedback from various sources, including employees, customers, and management. The study utilized an integrative literature review methodology to synthesize and evaluate previous literature, identifying the benefits that organizations can gain.

Horner, Jayawarna, Giordano, and Jones (2019) conducted a study on the effectiveness of university technology transfer, highlighting the importance of organizational support, such as the scale of Technology Transfer Office (TTO) support and the provision of incentives. The empirical results regarding the impact of these supports are mixed. Recent academic research and policy reviews emphasize the crucial role of strategic choices made by university managers in improving technology transfer effectiveness. This study aims to bridge these perspectives by applying Child’s strategic choice theory as a framework. Analyzing data from 115 UK universities collected through multiple waves of the HE-BCI Survey, the study finds that while organizational infrastructure is necessary for enhancing technology transfer, it is not sufficient on its own. It underscores the essential mediating role of strategic choice, indicating that alignment between the strategic choices made by university managers and the supporting organizational infrastructure explains variations in technology transfer effectiveness. The study also reveals that the relationship between strategic alignment and technology transfer effectiveness is influenced by the extent of strategic planning efforts, with universities that engage a broader range of faculty in strategic planning benefiting the most from this alignment.

Similarly, Gozali, Masrom, Zagloel, Haron, Garza-Reyes, Tjahjono, and Marie (2020) argue that measuring business process performance is a key concern for both faculty and industry players aiming for high productivity. Implementing a performance achievement framework for business incubator success factors ensures alignment with commercial strategies, thereby supporting high performance indicators in successful business incubator models. This research uses a quantitative approach, with data analyzed using IBM SPSS version 23 and Smart PLS version 3 statistical software. With a sample of 95 incubator managers from 19 universities in Indonesia, the study shows that the reputation of business incubator factors positively influences incubator performance. The research explores the relationship between incubator performance and success factors in Indonesia, finding that IT capabilities partially support performance; entry criteria directly enhance performance; mentoring networks improve performance, with infrastructure systems serving as a moderating factor; funding boosts performance, also moderated by good infrastructure; and university regulations and government support enhance performance, with credits and rewards acting as a moderating factor.

While, Dobija, Górska, Grossi, and Strzelczyk (2019) conducted a study to enhance the understanding of how performance measurement (PM) is utilized and by whom within universities. They gathered empirical data from four universities, allowing for a multilevel and comparative analysis grounded in neo-institutional theory. The study discusses its findings in relation to interdisciplinary literature on PM in the public sector. It reveals that PM practices have become increasingly prevalent across institutional, organizational, and individual levels within universities. Various types of PM are used with differing degrees of extent and scope, influenced by the involved actors. Universities often use PM ceremonially and symbolically to bolster their external image as research-focused institutions. The adoption of PM is shaped by both external factors, such as isomorphic pressures, and internal factors, including organizational and individual responses from university managers and academics. There are diverse attitudes and some resistance to PM within institutions. In universities with a local focus, PM for rational decision-making tends to be loosely linked with external accountability reporting. Additionally, the internal application of PM can also be symbolic.

Similarly, Agyemang and Broadbent (2015) examined the management control systems developed by universities and internal groups to oversee research within UK University Business and Management Schools. The study specifically analyzes how universities develop internal management control systems in response to externally imposed regulatory systems and proposes directions for future research. Using a middle-range approach, the research considers the UK Research Excellence Framework (REF) and prior Research Assessment Exercises. It employs various conceptual frameworks to analyze experiences and builds upon existing literature that highlights the negative effects of such performance measurement systems. The study found that internal management control systems developed by academics themselves amplify the controls imposed by the REF. While some academics accept these internal control systems, they also contribute to a shift away from previously held academic values.

In a related study, Karuhanga (2015) proposed a tool for assessing the implementation of strategic performance management (PM) by examining PM practices in public universities in Uganda. The study found that while strategic PM is present in these universities with the goal of enhancing quality, respondents generally disagreed that their institutions had an effective PM system, provided ongoing PM training for managers and staff, or had a formal process for units to provide feedback on goal attainment. The findings indicated that the evaluation of PM practices in universities could be based on five key areas: alignment of organizational vision, mission, strategy, and individual performance goals; staff involvement in PM implementation at the unit level; existence of an improvement plan; existence of a performance evaluation plan; and staff awareness and understanding of PM. While Guthrie, Manes-Rossi, Orelli, and Sforza (2024) conducted a review paper analyzing the literature on performance management and measurement (PMM) in universities over the past four decades. The analysis reveals that PMM has become a significant influence in universities, impacting their operations and redefining their identity. Research on PMM in universities has notably increased during this period. The paper provides an overview of published articles from these four decades, discussing content, themes, theories, methods, and impacts. It offers a foundation for examining past, present, and future research on university PMM. Future research directions include exploring PMM implementation strategies, interactions with government programs and external evaluations, and the role of various actors, particularly academics, in shaping PMM systems. Unlike traditional literature reviews, the structured approach used in this study offers insights into the evolution of the field and identifies potential areas for future research. This review encompasses a broader range of disciplines, including accounting, compared to previous reviews.

Whereas, Atwebembeire et al. (2018a, b) conducted a study to evaluate how performance monitoring affects the quality of teaching and research in private universities in Uganda. The study specifically investigated the impact of performance tracking, performance reviews, performance dialogue, and consequence management on teaching and research quality. Adopting a positivist approach and a cross-sectional survey design, the researchers selected four chartered private universities using disproportionate stratified random sampling based on foundation status. Data were gathered from 181 lecturers, 5 deans, 23 heads of department, 3 quality assurance officers, 3 senior officers from the National Council for Higher Education (NCHE), and 39 student leaders through questionnaires, interviews, document reviews, and observations. Descriptive statistics and regression analyses, complemented by content analysis, were used to interpret the data. The findings indicated that performance monitoring positively contributes to the quality of teaching and research. However, the study concluded that current staff performance monitoring practices in these universities are coercive and unsustainable for improving teaching and research quality. The authors recommend that university managers adopt more participatory performance monitoring mechanisms, where targets are collaboratively set, constructive feedback is given, and rewards are based on performance reviews. In a related study, Camilleri (2021a, b) examined managerialism and performance management within higher education. The research employed an inductive approach with semi-structured interviews involving academic staff. Utilizing the balanced scorecard (BSC) methodology, the study assessed participants’ views on their higher education institution’s (HEI) customer, internal, organizational capacity, and financial perspectives. The findings highlighted the strengths and limitations of using both financial and non-financial measures of the BSC to evaluate institutional performance and individual employee productivity. The study concluded that regular performance discussions with academic staff enable HEI leaders to better understand their institutions’ value-creating activities. It suggests that HEI leaders can leverage the BSC’s comprehensive framework as an effective tool for performance management, helping to regularly assess whether their institution is (i) providing inclusive, student-centered, high-quality education; (ii) producing impactful research; (iii) engaging with stakeholders; and (iv) enhancing its financial performance, among other positive outcomes.

Similarly, Grossi, Kallio, Sargiacomo, and Skoog (2020) investigated the challenges associated with the lack of a unified performance management system (PMS) that aligns with institutional strategic plans, which often results in unmet expectations. The study aimed to evaluate employees’ readiness for the implementation of a new PMS at a specific university and to identify obstacles, offering recommendations to address these issues. The study highlighted that universities frequently fail to develop PMSs tailored to their strategic plans, which should be extended to faculties and departments. Using a quantitative survey method, the researchers distributed a structured questionnaire to a sample of 150, receiving 108 completed responses, yielding a 72% response rate. A notable portion of respondents (34.3%) opposed the need for a PMS at their university, whereas 49.1% supported it, indicating a significant demand for such a system to mitigate manipulation and enforce strategic goals. The study identifies factors that may impede the successful implementation of PMS in universities and provides insights to help university leaders address these issues during planning. While, Mahdi, Nassar, and Almsafir (2019) contended that modern organizations have recognized the importance of acquiring and effectively managing knowledge to maintain a sustainable competitive advantage (SCA) in the marketplace. This suggests that organizational resources should encompass knowledge, which needs to be continuously nurtured and developed. For private colleges and universities, which are seen as investments in future business leaders, the knowledge management processes (KMP) at these institutions will inform future business strategies and competitiveness. However, there is some ambiguity about how the knowledge-based view (KBV) and resource-based view (RBV) relate to each other and the role of KMP in sustaining competitive advantage. This study aims to investigate how KMP can create SCA from the perspectives of KBV and RBV within the educational sector. A hypothesis was formulated, and a quantitative survey approach was used, with structural equation modeling (SEM) applied to analyze the relationships between study variables. The study involved 525 academic leaders from 44 private universities in Iraq. Findings indicated a significant link between KMP and SCA. To enhance SCA, private universities need to effectively generate, store, share, and apply knowledge, supported by the establishment of clear knowledge goals throughout the organization. This research adds to the existing literature on strategic knowledge management.

However, Alemiga and Kibukamusoke (2019a, b, c) highlighted that private universities (PUs) in Uganda, which emerged in the 1990s due to the privatization of higher education, face substantial challenges impacting educational quality. Issues such as admitting unqualified students and hiring underqualified faculty have undermined the quality of education. The National Council for Higher Education (NCHE), established by Act of Parliament No. 15 of 2011, is responsible for regulating and accrediting higher education institutions in Uganda to ensure they deliver quality, relevant, and standardized education. The NCHE oversees this through a quality assurance framework that includes both regulatory and institutional components. This study aimed to examine the factors influencing academic staff quality, with a focus on recruitment, development, promotion, and dismissal. Using total quality management theory, the research employed descriptive and case study methods, gathering data through interviews and observations. The findings indicated that while PUs have policies for managing academic staff, the enforcement, monitoring, and evaluation of these policies are inadequate, adversely affecting educational quality. Similarly, Bayasgalan (2015) conducted a study to examine the essential factors for effective employee performance in higher education institutions in Mongolia. The research proposed using the OCTAPACE culture model (Openness, Confrontation, Trust, Authenticity, Proactiveness, Autonomy, Collaboration, Experimentation) alongside workplace structure models to assess academic staff job satisfaction and commitment. While the OCTAPACE model is established in countries like India, Malaysia, and Western nations, it is relatively new in Mongolia. The study highlighted that employee job satisfaction and commitment play crucial roles in the performance of educational institutions. Analysis revealed that the OCTAPACE culture significantly affects job satisfaction and commitment, with workplace structure such as support from supervisors also having a substantial impact. The study involved a theoretical and empirical survey of 160 public and 143 private universities in Mongolia, analyzing data with SPSS 21 and Smart PLS 2.0 statistical software.

Despite the existence of multiple studies on performance monitoring in private universities (e.g., Kato & Tumwine, 2020; Nsubuga & Nalukwago, 2021), few have examined how specific monitoring mechanisms translate into measurable staff performance outcomes within Uganda’s chartered institutions. This study fills this gap by integrating quantitative and qualitative evidence to evaluate both the prevalence and developmental impact of performance monitoring systems.

### Theoretical framework

This study is anchored in two interrelated theoretical perspectives:

Henri Fayol’s Administrative Management Theory ( 1949): Fayol emphasizes five managerial functions: planning, organizing, commanding, coordinating, and controlling. In the context of performance monitoring, the controlling function is crucial in ensuring that staff activities are aligned with institutional objectives and that deviations are addressed promptly. The coordinating function further supports the integration of departmental monitoring activities with overarching university goals.

Victor Vroom’s Expectancy Theory ( 1964): This motivational theory suggests that individuals are motivated to perform when they believe their efforts will lead to high performance (expectancy), that performance will lead to desired outcomes (instrumentality), and that these outcomes are valued (valence). Performance monitoring, therefore, must be perceived as fair and consequential to encourage effort and engagement among academic staff.

While Fayol’s (1916) principles of management and Vroom’s (1964) expectancy theory are classical frameworks that have been extensively applied in organizational studies, their integration provides unique relevance to the Ugandan private university context. The centralized and mechanistic structures evident in many Ugandan institutions mirror Fayol’s emphasis on unity of command, division of work, and control, while Vroom’s focus on expectancy, instrumentality, and valence helps to explain how academic staff perceive motivation and rewards under performance monitoring regimes. By linking these two theories, the study highlights how organizational control mechanisms shape academic staff behavior and outcomes in resource-constrained environments.

Furthermore, this integration demonstrates originality in applying established theories to a less-explored geographical and institutional context. Unlike prior studies in Western or Asian higher education systems, Ugandan private universities face unique challenges such as limited funding, heavy reliance on tuition, and varied governance structures. Situating Fayol’s and Vroom’s frameworks within these realities enriches their explanatory power and enhances our understanding of academic staff performance in Africa.

While these theories provide a solid basis for examining performance monitoring and staff behavior, they have limitations in fully capturing contextual and dynamic aspects of higher education institutions. For instance, Fayol’s principles primarily focus on hierarchical structures, which may not fully address the flexibility and autonomy needed in academic settings, and Vroom’s framework may overlook external factors such as institutional culture or resource constraints. To address these gaps, future research could integrate complementary perspectives, including Herzberg’s motivation-hygiene theory, which distinguishes between intrinsic and extrinsic factors affecting job satisfaction, and contingency theory, which emphasizes that management practices should adapt to organizational and environmental conditions. By combining these theoretical lenses, scholars and practitioners can better design performance monitoring systems that are both effective and contextually appropriate.

Finally, we acknowledge that future research could extend this analysis by incorporating alternative perspectives such as Goal-Setting Theory ( Locke & Latham, 1990), which emphasizes specific and measurable targets, or Self-Determination Theory ( Deci & Ryan, 1985), which foregrounds intrinsic motivation. These frameworks could complement Fayol and Vroom by addressing the psychological and behavioral dimensions of academic work more comprehensively.

### Findings

**Types of Performance Monitoring used in private universities:** this is the second independent variable (IV), checking the performance monitoring methods for academic staff in private universities, it uses four different methods, namely evaluation methods, frequency of monitoring, feedback and reporting and use of technology.

**Evaluation Methods** this is the first area of performance monitoring methods for academic staff to be examined. The results are shown in Table 4.8.

### Interpretation and analysis of Table 1: Evaluation methods

**Table 1.** A table showing the evaluation methods.

S/No	Evaluation methods	F (%)	SD	D	U	A	SA	Mean	STD
B2.1	The university uses regular peer reviews to monitor academic staff performance.	F (%)	5(1.3)	26(6.7)	72(18.7)	160(41.5)	123(31.9)	3.96	0.94
B2.2	Student evaluations are used to assess teaching effectiveness.	F (%)	16(4.1)	17(4.5)	29(7.5)	200(51.8)	124(32.1)	4.03	0.97
B2.3	The university conducts annual performance appraisals for academic staff.	F (%)	18(4.7)	12(3.1)	39(10.1)	198(51.3)	119(30.8)	4.00	0.98
B2.4	Self-assessment is a part of the performance monitoring process.	F (%)	11(2.8)	29(7.5)	42(10.9)	203(52.6)	101(26.2)	3.92	0,96
B2.5	External reviews or audits are used to evaluate academic staff performance.	F (%)	13(3.4)	34(8.8)	42(10.9)	185(47.9)	112(29.0)	3.90	1.02
	**Grand mean**							3.96	

The table presents the frequency distribution and descriptive statistics of academic staff evaluations across five methods used by the university. The evaluation methods are assessed through a five-point Likert scale, ranging from Strongly Disagree (SD) to Strongly Agree (SA). Below is an interpretation of the findings:

The result of peer reviews (B2.1) showed 41.5% of respondents agreed, and 31.9% strongly agreed that the university uses regular peer reviews to monitor academic staff performance. A relatively small percentage (1.3%) strongly disagreed, suggesting broad support for peer reviews as an evaluation method. The mean score of 3.96 and standard deviation of 0.94 indicate a high level of agreement and moderate variation among responses. Student Evaluation data revealed that the majority (51.8% agreed and 32.1% strongly agreed) confirmed that student evaluations are used to assess teaching effectiveness. Only 4.1% strongly disagreed, showing that student evaluations are well-regarded as an evaluation tool. The mean score of 4.03, the highest in the table, reflects strong agreement, with a slightly higher variation (STD = 0.97) compared to peer reviews.

Annual performance appraisal is similar to student evaluations, 51.3% agreed 30.8% strongly agreed, and (7.8%) disagreed that the university conducts annual performance appraisals. The mean score of 4.00 signifies broad acceptance, with a slightly higher variation (STD = 0.98) than student evaluations and peer reviews. Self-Assessment (B2.4): Over half (52.6% agreed) that self-assessment is part of the performance monitoring process, while 26.2% strongly agreed. A small percentage (2.8% strongly disagreed), indicating limited resistance to self-assessments. The mean score of 3.92 is slightly lower than the other methods but still indicates overall agreement. The standard deviation of 0.96 suggests moderate variation. External reviews or audits showed47.9% agreed and 29.0% strongly agreed that external reviews or audits are used for evaluating academic staff performance. With 3.4% strongly disagreeing, this method has the highest standard deviation (STD = 1.02), indicating slightly more diverse opinions compared to other methods. The mean score of 3.90 is the lowest among the five methods but still reflects overall agreement.

The grand mean of 3.96 indicates that, on average, respondents agree with the effectiveness and implementation of these evaluation methods. The mean scores range from 3.90 to 4.03, with student evaluations being the most positively rated method. Additionally, the standard deviations (ranging from 0.94 to 1.02) suggest moderate variation in responses across all methods. The table highlights a generally positive perception of the university’s evaluation methods, with room for targeted improvements in specific areas.

**Frequency of Monitoring** this is the second area of performance monitoring methods for academic staff to be examined. The results are shown in Table 4.9.

### Analysis and interpretation of data

Table 2 presents data on the frequency and adequacy of monitoring practices and their perceived impact on performance improvement. It is based on a survey scale from 1 (Strongly Disagree - SD) to 5 (Strongly Agree - SA). Here is the analysis and interpretation of performance monitoring regularity (B2.6), The majority agree (44.6%) and strongly agree (35.8%) that performance monitoring is conducted regularly (daily, weekly, monthly, quarterly, and annually). Mean: 4.03, STD: 1.02, respondents agree that performance monitoring is routinely conducted. However, the STD of 1.02 suggests some variability in how regularly respondents perceive this practice to be implemented, indicating strong implementation of structured performance monitoring.

**Table 2.** A table showing the Frequency of Monitoring.

S/No	Frequency of Monitoring	F (%)	SD	D	N	A	SA	Mean	STD
B2.6	Performance monitoring is conducted regularly (Daily, Weekly, monthly, quarterly, and annually).	F (%)	18(4.7)	14(3.6)	44(11.4)	172(44.6)	138(35.8)	4.03	1.02
B2.7	The frequency of monitoring is adequate to ensure continuous improvement.	F (%)	8(2.1)	26(6.7)	48(12.4)	203(52.6)	101(26.2)	3.94	0.92
B2.8	Informal performance checks are conducted frequently.	F (%)	12(3.1)	26(6.7)	45(11.7)	171(44.3)	132(34.2)	4.00	0.95
B2.9	Frequent feedback sessions are organized to discuss progress and areas for development.	F (%)	7(1.8)	29(7.5)	59(15.3)	179(46.4)	112(29.0)	3.93	0.95
B2.10	Regular monitoring helps identify challenges early and implement corrective actions promptly.	F (%)	10(2.6)	31(8.0)	62(16.1)	187(48.4)	96(24.9)	3.85	1.01
	**Grand mean**							3.95	

The adequacy of monitoring frequency checks shows that the majority (52.6%) strongly agree (26.2%) that monitoring frequency ensures continuous improvement. Mean: 3.94, STD: 0.92, most respondents feel the frequency of monitoring is adequate for continuous improvement, with relatively less variation (lower STD), suggesting perceived sufficiency of monitoring schedules to foster improvements. Informal performance results showed that agreement is high, with 44.3% agreeing and 34.2% strongly agreeing that informal performance checks are frequent. Mean: 4.00, STD: 0.95, shows there is an agreement that informal performance checks are conducted frequently, with moderate consistency in responses.

Feedback sessions show a significant proportion of respondents agree (46.4%) and strongly agree (29.0%) that feedback sessions are regularly conducted, while (9.3%) disagree. With a mean of 3.93, STD: 0.95, it shows that respondents agree that feedback sessions are organized regularly, indicating structured feedback sessions are valued as tools for progress evaluation and development. Finally, monitoring for early problem identification shows that agreement is moderate to high, with 48.4% agreeing and 24.9% strongly agreeing, while 18% disagree on the effectiveness of regular monitoring for early challenge identification and corrective action. With a mean of 3.85 and std of 1.01, it shows regular monitoring is acknowledged as effective for early identification of challenges, though responses show slightly more variability compared to other items, suggesting monitoring contributes to proactive problem-solving. The grand mean of 3.95indicates a positive perception of the regularity and adequacy of monitoring practices in ensuring continuous improvement and addressing challenges effectively.

### Interpretation of the pie chart

The chart represents the distribution of responses to the statement: “Performance monitoring is conducted regularly (e.g., quarterly, annually).” The data shows the percentage of respondents selecting each of the following options: strongly disagree (blue), disagree (green), neutral (beige), agree (purple), and strongly agree (yellow). Most respondents expressed agreement (44.56% Agree) and strong agreement (35.75% Strongly Agree) with the statement. Combined, 80.31% of respondents positively perceive the regularity of performance monitoring. Neutral Responses a smaller proportion (11.40%) of respondents remained neutral, indicating they neither agreed nor disagreed. This could suggest uncertainty or mixed experiences with the monitoring practices. Negative Responses, only a small minority disagreed (3.63%) or strongly disagreed (4.66%), making up 8.29% of respondents, this reflects minimal dissatisfaction with the regularity of performance monitoring. The chart reflects a strong consensus among respondents that performance monitoring is conducted regularly. The small percentage of neutral and negative responses suggests room for improvement in ensuring the consistency of monitoring practices for all stakeholders.

**Figure 1.**
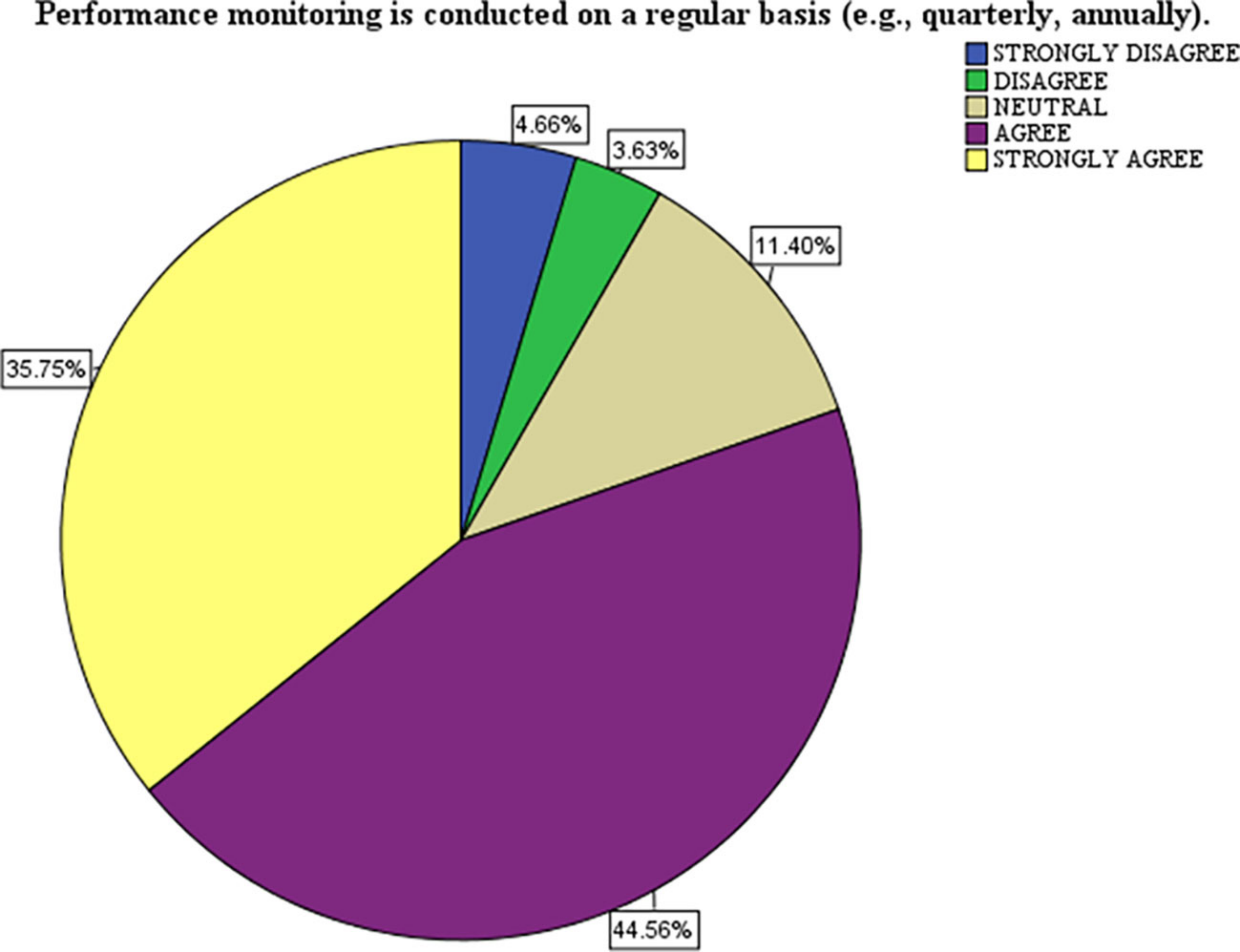
A Pie Chart Showing the Visual Representations of Performance Monitoring.

### Interpretation of feedback and reporting data

Table 3 evaluates academic staff views of feedback and reporting practices related to performance monitoring. The responses range from Strongly Disagree (SD) to Strongly Agree (SA), with the mean and standard deviation providing insights into agreement levels and response variability. Reporting Mechanisms data showed that a majority of respondents agree (48.4%) and strongly agree (34.2%), while (8.8%) disagree, that reporting mechanisms are in place to document staff achievements and challenges. The mean is 4.03 and the std is 1.02, responses are relatively consistent, indicating agreement on establishing reporting mechanisms. This shows strong confidence in the existence of structured reporting systems. Performance Reports responses showed that most respondents agree (46.4%) and strongly agree (29.0%) while (11.4) disagree that academic staff receive regular performance reports. The mean is 3.87, and STD is 1.09Suggest regular reporting practices, though room for improvement exists due to 13.2% neutrality and 11.4% disagreement.

**Table 3.** A table showing the Feedback and Reporting.

S/N	Feedback and Reporting	F (%)	SD	D	N	A	SA	Mean	STD
	Reporting mechanisms are established to document staff achievements and challenges.	F (%)	20(5.2)	14(3.6)	33(8.5)	185(48.4)	132(34.2)	4.03	1.02
B2.12	Academic staff receive performance reports regularly.	F (%)	25(6.5)	19(4.9)	51(13.2)	179(46.4)	112(29.0)	3.87	1.09
B2.13	There is a formal process for discussing performance results with academic staff.	F (%)	20(5.2)	44(11.4)	41(10.6)	177(45.9)	104(26.9)	3.78	1.12
B2.14	Feedback from performance monitoring is used to support professional development.	F (%)	19(4.9)	50(13.0)	51(13.2)	130(33.7)	136(35.2)	3.81	1.19
B2.15	The feedback and reporting process is transparent, promoting accountability and trust.	F (%)	25(6.5)	58(15.0)	43(11.1)	145(37.6)	115(29,8)	3.69	1.23
	**Grand mean**							3.84	

Furthermore, Formal Discussion of Performance Results (B2.13): A significant proportion agrees (45.9%) and strongly agree (26.9%) that formal processes exist for discussing performance. However, 11.4% disagreement and 10.6% neutrality reflect variability in the perception of these discussions. Mean: 3.78, STD: 1.12, Responses are more spread out, indicating a wider range of opinions about the balance between central control and departmental autonomy. Similarly, feedback for professional development responses revealed that most respondents agree (33.7%) and strongly agree (35.2%) that feedback is used for professional development. Nonetheless, higher neutrality (13.2%) and disagreement (17.9%) suggest the need for clearer feedback alignment with development goals. Mean: 3.81, STD: 1.19 meaning responses show noticeable variability, indicating varied perceptions of how feedback supports professional development.

Finally, Transparency and Accountability in Feedback (B2.15): While 67.4% (combined agree and strongly agree) perceive the feedback process as transparent, 15% disagree and 11.1% are neutral, indicating potential gaps in perceived accountability and trust. The Mean is 3.69, STD: of 1.23, meaning the highest variability reflects diverse views on transparency and accountability. The Grand Mean is 3.84showing that the overall perception of feedback and reporting mechanisms is positive, with most means hovering near 4.00, reflecting general agreement with the practices in place. Standard deviations suggest moderate variability in perceptions, indicating differing experiences or inconsistencies.

### Analysis of Table 4

**Table 4.** A table use of technology.

S/N	Use of technology	F (%)	SD	D	N	A	SA	Mean	STD
B2.16	Technology provides academic staff with tools for efficient communication and collaboration.	F (%)	22(5.7)	20(5.2)	30(7.8)	191(49.5)	123(31.9)	3.97	1.06
B2.17	It enables easy access to educational resources and research materials.	F (%)	17(4.4)	21(5.4)	62(16.1)	173(44.8)	113(29.3)	3.89	1.03
B2.18	Online platforms are used for collecting and analyzing performance data.	F (%)	34(8.8)	36(9.3)	37(9.6)	182(47.2)	97(25.1)	3.70	1.20
B2.19	Technology enhances the accuracy and efficiency of performance monitoring.	F (%)	14(3.6)	43(11.1)	50(13.0)	153(39.6)	126(32.6)	3.87	1.10
B2.20	The university uses digital tools to monitor academic staff performance.	F (%)	9(2.3)	16(4.1)	21(5.4)	205(53.1)	135(35.0)	4.14	0.87
	**Grand mean**							3.91	

The table provides detailed insights into academic staff perceptions of technology usage, categorized into five response levels: Strongly Disagree (SD), Disagree (D), Neutral (N), Agree (A), and Strongly Agree (SA). Here’s an analysis that includes all these categories:

Concerning whether technology provides academic staff with tools for efficient communication and collaboration, most respondents agree (81.4%), while only (10.7%). A mean of 3.97 indicates that most staff agree or strongly agree that technology supports communication and collaboration effectively. STD of (1.06) moderate variability shows some differences in opinion. The result of the technology enabling easy access to educational resources and research materials showed that most respondents agree (74.1%) and (9.8%) disagree. Mean (3.89): indicates agreement that technology facilitates access to resources, but the higher percentage of “Uncertain” responses (16.1%) highlights some ambiguity, and STD of (1.03) low variability suggests consistent responses.

Similarly, the data on online platforms used for collecting and analyzing performance data revealed that (72.3%) agree while (18.1%) disagree. (3.70) lower mean indicates that while many agree, a significant portion of respondents (27.7%) disagreed or were uncertain about using online platforms for performance data. STD (1.20): The highest variability reflects diverse opinions, suggesting inconsistencies in implementation. Results on whether technology enhances the accuracy and efficiency of performance monitoring showed that (72.2%) agree and (14.7%) disagree. With a mean of 3.87 indicating moderate agreement on the accuracy and efficiency of technology in performance monitoring, 27.7% of respondents were uncertain or disagreed and STD (1.10) moderate variability suggests some differences in perception.

The university uses digital tools to monitor academic staff performance results showed that (88.1%) agree, and only (6.4%) disagreed, (5.4%) were neutral. Mean (4.14): The highest mean reflects strong agreement and confidence in the university’s use of digital tools for monitoring performance. STD (0.87) the lowest variability indicates strong consensus among respondents. The grand mean (3.91) reflects moderate to strong agreement across all statements, showing positive perceptions overall. Grand STD (~1.05) shows moderate variability across responses suggesting differing perceptions or experiences among academic staff.

### Overall

Academic staff generally recognize the benefits of technology, but addressing gaps in specific areas, such as data collection platforms and resource accessibility, can further improve satisfaction and productivity.

Objective Two: To find out the types of performance monitoring used in private chartered universities in Western Uganda.

Quantitative Results: Survey findings indicated:

59% agreed that student evaluations were conducted regularly.

54% acknowledged peer review processes.

47% reported receiving regular supervisory feedback.

Only 38% considered the performance monitoring system as consistent and fair.

Pearson correlation analysis revealed a moderate positive relationship (r = 0.48, p < 0.05) between the perceived effectiveness of performance monitoring and self-reported performance levels. Furthermore, regression analysis showed that performance monitoring practices significantly predicted academic staff performance, with an R ^2^ value of 0.213 (F(1, 184) = 49.79, p < 0.001), indicating that approximately 21.3% of the variance in performance was explained by the effectiveness of monitoring systems.

Qualitative Results: Ten thematic quotes from academic deans:

“Performance monitoring is mostly done through end-of-semester student evaluations, but the feedback is rarely followed up.” (Dean 1, DU PT1-31)

“Peer reviews are formalities, not developmental tools. They’re done for compliance.” (Dean 3, DU PT3-49)

“We do monitor performance, but the outcomes are not linked to professional development or promotions.” (Dean 5, DU PT5-44)

“Supervisors are expected to monitor staff, but some lack training in how to conduct meaningful evaluations.” (Dean 7, DU PT7-59)

“Staff become demotivated when monitoring feels punitive rather than supportive.” (Dean 9, DU PT9-66)

“The structure is there, but its implementation depends on who is in charge. Some Deans are proactive, others just sign off forms.” (Dean 2, DU PT2-33)

“In many departments, monitoring happens only when there’s a crisis or when complaints arise from students.” (Dean 4, DU PT4-41)

“There’s a sense that performance evaluation is for punishment, especially when the reports go to HR without proper discussion with staff.” (Dean 6, DU PT6-53)

“Monitoring could be useful, but without incentives or recognition, it’s hard to motivate staff to take it seriously.” (Dean 8, DU PT8-61)

“When staff see no change regardless of performance, they begin to ignore the process.” (Dean 10, DU PT10-70)

The study found that mechanistic organizational structures dominate private chartered universities in Western Uganda. Quantitative results show that 72% of respondents reported centralized decision-making and high levels of formalization, while 68% indicated that performance monitoring occurs frequently but focuses primarily on compliance rather than staff development. These findings are reinforced by qualitative insights: one participant stated, “Decisions are made at the top, and we follow instructions with little room for flexibility” (Participant 14, University B), and another noted, “Performance evaluations focus more on ticking boxes than on improving our teaching skills” (Participant 7, University C).

The integration of quantitative and qualitative data highlights consistent themes of centralization, formalization, and compliance-oriented monitoring. While performance monitoring is systematic, the focus on procedural adherence may limit motivation and professional growth. The results indicate that monitoring practices are well established but not always aligned with staff development objectives. These findings are reflected across survey results, interview quotes, and document reviews, demonstrating strong consistency and coherence in the data.

Interpretation: The data affirm that performance monitoring systems exist but are often inconsistently implemented and lack a developmental focus. The absence of clear links between monitoring results and staff rewards or growth opportunities demotivates staff. From Fayol’s perspective, this represents a failure in managerial control and coordination, resulting in ineffective monitoring. Vroom’s theory further clarifies the motivational deficit: when staff do not believe monitoring leads to meaningful rewards, their engagement diminishes.

## Discussion

The findings indicate that mechanistic structures and compliance-oriented performance monitoring significantly shape academic staff performance. Highly centralized decision-making and formalized rules, as identified in 72% of respondents, limit staff autonomy and flexibility, which can negatively affect motivation and engagement ( Silaji, Bagiwa, & Muhammad, 2025b; Shimaneni, 2025). While Qualitative insights confirm that monitoring often emphasizes procedural compliance over developmental feedback, reflecting challenges observed in similar African higher education contexts ( Netshandama, 2023). The dominance of mechanistic structures in performance monitoring, where evaluations focus on compliance rather than developmental outcomes, reflects challenges observed in Ugandan private universities ( Atwebembeire & Malunda, 2019).

By integrating quantitative and qualitative results, it is clear that while monitoring ensures accountability, it may not effectively foster professional growth. These findings highlight the importance of context-sensitive performance monitoring frameworks that balance oversight with staff development. Mixed-methods evidence supports the notion that combining structured evaluation with supportive feedback can enhance performance outcomes ( Ezzeddine, Otaki, Darwish, & AlGurg, 2023; Albaroudi & Alenezi, 2025).

The study reveals that while performance monitoring is institutionalized, its effectiveness is hampered by poor implementation and lack of alignment with staff development. As Armstrong (2020) notes, monitoring should drive improvement, not merely compliance. Fayol’s theory highlights that effective management demands consistent application of control and coordination, both of which are lacking in the studied institutions. Vroom’s theory shows that performance monitoring, when unlinked to motivation, fails to drive performance. These shortcomings reduce the perceived value of monitoring systems and contribute to academic staff disengagement.

The present study reveals that performance monitoring tools such as student evaluations, peer reviews, and supervisory assessments are implemented to varying degrees across private chartered universities in Western Uganda. However, their inconsistent application and weak alignment with professional development goals limit their effectiveness.

These findings align with those of Atwebembeire et al. (2018a, b), who concluded that performance monitoring practices in private universities are often coercive and unsustainable, failing to enhance quality teaching and research effectively. Their study emphasized the need for participatory-oriented performance monitoring mechanisms, where targets are agreed upon, constructive feedback is provided, and staff are rewarded based on performance reviews.

Similarly, Birungi et al. (2024) found that effective management practices, including performance appraisals and development opportunities, positively influence academic staff performance in private universities. Their research underscores the importance of aligning performance monitoring with professional development to promote ethical standards and organizational identity among staff.

However, our study diverges from these findings by highlighting the inconsistent implementation of performance monitoring tools and their limited developmental integration. While previous studies advocate for participatory approaches, our findings suggest that resource constraints, institutional culture, and policy environments may hinder the adoption of such practices.

For instance, Ssentamu (2019) noted that the National Council for Higher Education (NCHE) in Uganda faces challenges in enforcing regulations and minimum standards due to inadequate professional and technical capacity. This lack of capacity may contribute to the inconsistent application of performance monitoring practices across institutions.

Furthermore, the study by Mbyemeire et al. (2024) emphasized that monitoring helps identify implementation challenges that may hinder the achievement of intended outcomes. They recommend that private higher academic institutional managers should augment the budget for monitoring to enhance accountability and improve program implementation.

Related to the above, this study revealed that performance monitoring tools—such as student evaluations, peer reviews, and supervisory assessments—are implemented to varying degrees across private chartered universities in Western Uganda. However, their inconsistent application and weak alignment with professional development goals limit their effectiveness.

Several factors contribute to the prevalence and variability of these performance monitoring practices:

**Resource Constraints**: Many private universities operate under limited financial and human resources, which hampers the consistent implementation of comprehensive performance monitoring systems. This scarcity affects the ability to conduct regular evaluations and provide feedback mechanisms essential for staff development.

**Institutional Culture**: The organizational culture within some universities may not prioritize performance monitoring, viewing it as a punitive measure rather than a developmental tool. This perception can lead to resistance among staff and a lack of engagement with evaluation processes.

**Policy Environments**: The absence of clear policies and guidelines from regulatory bodies on performance monitoring practices can result in ad hoc and inconsistent application across institutions. Without standardized frameworks, universities may struggle to implement effective monitoring systems. These factors underscore the need for a more structured and supportive approach to performance monitoring that considers the unique challenges faced by private universities in the region. Monitoring and evaluating academic staff performance can be complex due to contextual and institutional constraints ( Netshandama, 2023). This aligns with our findings that mechanistic structures in private universities may limit flexibility in performance assessment.

This study set out to evaluate the types of performance monitoring employed in private chartered universities in Western Uganda and to assess their effectiveness in influencing academic staff performance. The findings demonstrate that while performance monitoring tools such as student evaluations, peer reviews, annual appraisals, and supervisory assessments are present, their effectiveness is undermined by inconsistency, weak feedback mechanisms, and limited integration into staff development initiatives.

From a theoretical standpoint, Fayol’s Administrative Management Theory highlights the importance of coordination and control in aligning individual performance with institutional objectives. Our findings suggest that while control mechanisms exist, coordination is weak, leading to fragmented monitoring practices across departments. Similarly, Vroom’s Expectancy Theory underscores that performance monitoring can only enhance motivation when staff perceive a clear link between effort, performance, and rewards. In this study, staff reported that monitoring outcomes were rarely tied to tangible incentives, professional growth, or career advancement, which explains the moderate correlation (r = 0.48) between monitoring and performance. Without perceived instrumentality, monitoring risks being viewed as punitive rather than developmental.

These results align with Atwebembeire et al. (2018a, b), who found that performance monitoring practices in Ugandan private universities were often coercive and unsustainable, focusing on compliance rather than capacity-building. Similarly, Nsubuga and Nalukwago (2021) reported that monitoring enhances productivity only when coupled with transparent metrics and integration into professional development programs. Our study adds nuance by showing that even where tools exist, inconsistent application erodes trust and diminishes impact, echoing the concerns of Waiswa and Kiggundu (2022) regarding the lack of uniform monitoring standards and inadequate feedback.

The findings also resonate with Birungi et al. (2024), who emphasized that effective management practices, including performance monitoring, must be tied to developmental opportunities and ethical standards to sustain organizational identity. In our case, staff expressed dissatisfaction when monitoring was reduced to form-filling exercises, confirming that the absence of developmental focus demotivates academic staff. This reflects Armstrong’s (2020) assertion that performance monitoring should drive improvement and engagement rather than serve as a compliance exercise.

Contrastingly, studies in other contexts (e.g., Kagina & Mwesigwa, 2022; Mugisha & Okello, 2021) found that robust monitoring systems reduced staff turnover and improved productivity by linking evaluation results to support mechanisms. The divergence from our findings may be attributed to resource constraints in Ugandan private universities, where limited budgets hinder the consistent implementation of structured monitoring and feedback systems. Furthermore, as Ssentamu (2019) notes, the limited enforcement capacity of the National Council for Higher Education contributes to uneven institutional practices.

This study therefore underscores that the problem is not the absence of performance monitoring mechanisms, but rather their poor operationalization. Monitoring is often perceived as punitive, inconsistent, and disconnected from professional growth, which undermines its motivational potential. Addressing these gaps requires reorienting performance monitoring from a control-focused activity to a participatory and developmental process.

Collectively, these findings underscore that performance monitoring in Ugandan private universities functions more as a compliance mechanism than as a developmental tool. To enhance effectiveness, universities should institutionalize regular feedback sessions linked to professional development programs, strengthen supervisor training in evaluation techniques, and integrate performance data into promotion and reward systems. Policymakers should also standardize performance monitoring guidelines across private institutions to ensure fairness and accountability.

### Practical implications

The findings of this study have several practical implications for stakeholders in the higher education sector:

**University Administrators**: There is a need to foster a culture that views performance monitoring as a tool for professional growth rather than solely for accountability. Administrators should invest in training programs that equip academic staff with the skills to engage constructively with evaluation processes.

**Policy-Makers **: Regulatory bodies should develop and disseminate clear guidelines on performance monitoring practices to ensure consistency across institutions. Policies should emphasize the developmental aspects of performance evaluations and provide support for capacity-building initiatives.

**Educators**: Academic staff should be encouraged to participate actively in the design and implementation of performance monitoring tools. Engagement in these processes can enhance their relevance and effectiveness, leading to improved teaching and research outcomes.

By addressing these areas, stakeholders can work collaboratively to enhance the effectiveness of performance monitoring systems, ultimately improving academic staff performance and institutional quality.

### Limitations and future research

While this study provides valuable insights into performance monitoring practices in private chartered universities in Western Uganda, certain limitations must be acknowledged:

**Geographic Scope**: The study focuses on universities within a specific region, which may limit the generalizability of the findings to other contexts.

**Self-Reported Data**: Reliance on self-reported data from academic staff and administrators may introduce biases, as participants might present socially desirable responses.

**Sampling Technique**: The use of stratified random sampling, while ensuring representation, may not capture the full diversity of experiences across all private universities.

### Theoretical implications

This study underscores the necessity of integrating structural and motivational theories in designing performance monitoring systems. Fayol’s administrative functions provide the framework for establishing formalized, coordinated, and controlled monitoring systems. Vroom’s expectancy theory explains how these systems should be aligned with motivational outcomes such as promotions and rewards to foster staff engagement. Combining both theories provides a comprehensive model for understanding and improving academic staff performance through effective monitoring.

### Practical implications of the study

The findings of this study carry both theoretical and practical implications for higher education institutions, particularly in developing contexts. Theoretically, our results underscore the relevance of integrating control and motivation theories when designing performance monitoring systems, as these frameworks help explain how organizational structures and monitoring practices influence academic staff performance. Practically, the study provides guidance for policy adaptations in low-resource universities, recommending context-sensitive performance monitoring mechanisms that balance formal oversight with staff autonomy and motivation. By tailoring monitoring frameworks to institutional capacity and the unique needs of academic staff, universities can enhance performance outcomes while promoting a supportive and accountable work environment. Strengthening performance monitoring to move beyond rigid, control-driven approaches toward participatory and developmental models could address the structural weaknesses identified in earlier Ugandan studies ( Atwebembeire & Malunda, 2019).

Previous research emphasizes that academic staff perceptions of performance monitoring significantly influence their productivity and motivation ( Silaji et al., 2025b; Shimaneni, 2025). These studies highlight that well-designed monitoring frameworks are critical for improving staff performance in low-resource higher education contexts.

## Conclusion

Performance monitoring in private chartered universities in Western Uganda is structurally present but operationally flawed. The inconsistency in implementation and the lack of developmental orientation reduce its effectiveness. Applying Fayol’s principles of coordination and control, and Vroom’s motivational expectations, reveals that both system design and outcome alignment are essential for effective staff performance monitoring.

In summary, while our findings corroborate previous research on the positive relationship between performance monitoring and academic staff performance, they also highlight the need for consistent implementation and integration of developmental aspects into performance monitoring practices. Addressing resource constraints, fostering a supportive institutional culture, and strengthening policy frameworks are essential steps toward enhancing the effectiveness of performance monitoring in private universities.

In conclusion, while performance monitoring systems exist in private chartered universities in Western Uganda, their developmental potential remains underutilized. Strengthening the link between monitoring outcomes and staff capacity-building will enhance motivation, accountability, and institutional quality. Future interventions should emphasize supervisor training, feedback utilization, and the integration of monitoring outcomes into human resource and professional development frameworks.

### Recommendations

Train academic leaders in constructive performance monitoring techniques.

Standardize monitoring practices across departments to ensure fairness.

Link monitoring outcomes to tangible rewards, promotions, and development opportunities.

Encourage participatory monitoring systems to foster staff buy-in.

Reframe monitoring from punitive assessment to developmental feedback.

Future research should consider expanding the geographic scope to include other regions and incorporate longitudinal designs to assess changes over time. Additionally, employing mixed-method approaches that include observational data could provide a more comprehensive understanding of performance monitoring practices.

## Ethical approval statement

This study received ethical approval from the **Research Ethics Committee of Kampala International University**, Uganda. The approval was granted on **September 6** ^**th**^ **2024**, with the reference number **KIU-2024-292**. The ethics committee approved the research protocol, participant recruitment procedures, and data protection measures. Also on the same date, Research Ethics Committee approved the use of **verbal informed consent** due to the minimal risk nature of the study and literacy considerations among some participants. The manuscript has been updated to include the ethical approval date. The Uganda National Council for Science and Technology (UNCST) granted ethical approval on **8** ^**th**^ **October 2024**, under national approval number **SS3145ES**. uncst.go.ug

## Data Availability

Repository name: *Evaluating the Types of Performance Monitoring Used in Private Chartered Universities in Uganda.* https://doi.org/10.17605/OSF.IO/79KWP ( Silaji et al., 2025a). Data are available under the terms of the Creative Commons Attribution 4.0 International license (CC-BY 4.0).
