# Supplementary material for: Evaluating the Types of Performance Monitoring Used in Private Chartered Universities in Uganda: A Mixed-Methods
Source: F1000Res. 2025 Oct 23;14:675. Originally published 2025 Jul 8. [Version 3] doi: 10.12688/f1000research.166395.3 (PMC12658881; doi:10.12688/f1000research.166395.3)
Supplement: Peer review history

# Reviewer report: Reviewer response for version 3

**DOI:** 10.5256/f1000research.189714.r426294

**Published:** 2025-11-07

**Authors:** Issah Iddrisu

**Affiliations:** 1 University for Development Studies, Tamale, Ghana

no further comments

Is the work clearly and accurately presented and does it cite the current literature?

Partly

If applicable, is the statistical analysis and its interpretation appropriate?

Yes

Are all the source data underlying the results available to ensure full reproducibility?

Yes

Is the study design appropriate and is the work technically sound?

Yes

Are the conclusions drawn adequately supported by the results?

Yes

Are sufficient details of methods and analysis provided to allow replication by others?

Partly

Reviewer Expertise:

Public Management and Organisational behaviour

I confirm that I have read this submission and believe that I have an appropriate level of expertise to confirm that it is of an acceptable scientific standard.

# Reviewer report: Reviewer response for version 2

**DOI:** 10.5256/f1000research.187266.r416256

**Published:** 2025-10-13

**Authors:** Issah Iddrisu

**Affiliations:** 1 University for Development Studies, Tamale, Ghana

**1. Is the work clearly and accurately presented, and does it cite the current literature?**

**Partly.** The manuscript is generally clear and well organized, with a coherent abstract and introduction. The authors present a logical argument for studying performance monitoring in private universities and effectively connect it to broader educational management theories. However, the literature review is overly lengthy and occasionally repetitive, particularly where earlier studies are restated with similar details. While the revised version includes recent (2023–2025) references, some sources are summarised rather than critically analysed. The linkage between cited works and the study’s research gap could be made more explicit.

*Recommendation:* Condense the literature review by focusing on the most relevant and recent studies. Clearly articulate how the present study builds on or diverges from prior work.

**2. Is the study design appropriate and is the work technically sound?**

**Yes, generally.** The concurrent triangulation mixed-methods design is appropriate for the study’s objectives. Quantitative and qualitative data collection from academic staff and deans provides depth and breadth. Sampling procedures are adequately described, and the rationale for combining Fayol’s Administrative Management Theory and Vroom’s Expectancy Theory is sound. The mixed-methods integration, however, could be better illustrated—especially how qualitative themes were compared or contrasted with quantitative results.

*Recommendation:* Include a clearer explanation (possibly a diagram or table) showing how qualitative and quantitative findings were integrated during analysis.

**3. Are sufficient details of methods and analysis provided to allow replication by others?**

**Partly.** Data collection instruments, sampling techniques, and analytical methods (SPSS, NVivo, correlation, regression) are adequately described. However, the article could better explain how validity and reliability tests were conducted. For example, the actual Cronbach’s alpha coefficients or sample thematic codes are not presented. The regression model summary is given, but assumptions such as multicollinearity or normality are not discussed.

*Recommendation:* Provide the reliability coefficients and a sample of the coding framework or interview themes. Briefly address statistical assumptions to strengthen reproducibility.

**4. If applicable, is the statistical analysis and its interpretation appropriate?**

**Yes, but interpretation could be deepened.** The correlation (r = 0.48, p < 0.05) and regression (R² = 0.213) results are correctly reported and interpreted as showing a moderate positive relationship. However, the discussion could elaborate more on practical significance—what does 21% explained variance mean for policy or practice? Additionally, the connection between quantitative outcomes and qualitative quotes should be emphasised more directly.

*Recommendation:* Discuss effect sizes in practical terms and highlight triangulation between numeric trends and interview insights.

**5. Are all ethical considerations addressed and adequately described?**

**Yes.** Ethical approval from institutional review boards and the Uganda National Council for Science and Technology is clearly stated, with reference numbers. The informed consent process (written and verbal) is well explained and aligns with research ethics standards for educational studies.

**6. Are the conclusions supported by the results?**

**Yes, but could be more focused.** The conclusions are supported by both data strands and are consistent with the study’s objectives. However, the section could more directly highlight implications for policy, management training, or institutional reform. The discussion sometimes re-summarises findings instead of synthesising them.

*Recommendation:* Emphasise key actionable outcomes—such as integrating performance monitoring results into professional development programs—and clarify limitations.

**7. Is the manuscript well-structured and presented in a professional manner?**

**Yes.** The paper follows a logical academic structure with clear headings and tables. Some tables could be summarised for brevity, and minor grammatical issues (e.g., redundant phrases and inconsistent tense) should be addressed before publication.

**Overall Assessment**

 This paper provides valuable empirical insights into performance monitoring practices in Ugandan private universities. With moderate revisions to streamline the literature review, clarify methodological details, and strengthen the discussion, it would meet the standards.

Is the work clearly and accurately presented and does it cite the current literature?

Partly

If applicable, is the statistical analysis and its interpretation appropriate?

Yes

Are all the source data underlying the results available to ensure full reproducibility?

Yes

Is the study design appropriate and is the work technically sound?

Yes

Are the conclusions drawn adequately supported by the results?

Yes

Are sufficient details of methods and analysis provided to allow replication by others?

Partly

Reviewer Expertise:

Public Management and Organisational behaviour

I confirm that I have read this submission and believe that I have an appropriate level of expertise to confirm that it is of an acceptable scientific standard, however I have significant reservations, as outlined above.

# Reviewer report: Reviewer response for version 2

**DOI:** 10.5256/f1000research.187266.r411880

**Published:** 2025-09-27

**Authors:** M Mukhibat Syaufa

**Affiliations:** 1 Institut Agama Islam Negeri (IAIN) Ponorogo, Ponorogo, Indonesia

This article also makes practical contributions to university development policy and management practice. With its solid methodology, analysis, and presentation, this article is highly suitable for acceptance for indexing without further revision.

Is the work clearly and accurately presented and does it cite the current literature?

No

If applicable, is the statistical analysis and its interpretation appropriate?

Yes

Are all the source data underlying the results available to ensure full reproducibility?

Yes

Is the study design appropriate and is the work technically sound?

Yes

Are the conclusions drawn adequately supported by the results?

Yes

Are sufficient details of methods and analysis provided to allow replication by others?

Yes

Reviewer Expertise:

My research focus is on education, Islamic education management, education evaluation, teacher education

I confirm that I have read this submission and believe that I have an appropriate level of expertise to confirm that it is of an acceptable scientific standard.

# Response

**Published:** 2025-09-29

**Authors:** Turyamureeba silaji

**Affiliations:** Foundations, Kampala International University - Western Campus, Bushenyi, Western Region, Uganda

**Final Note of Appreciation**

 We sincerely thank the reviewer for the constructive feedback and for recognizing the strengths of our manuscript. We are especially encouraged by the acknowledgment that:

*“This article also makes practical contributions to university development policy and management practice. With its solid methodology, analysis, and presentation, this article is highly suitable for acceptance for indexing without further revision.”*

 This positive evaluation affirms the rigor and relevance of our study, and we are deeply grateful for the reviewer’s thoughtful assessment and endorsement.

# Reviewer report: Reviewer response for version 1

**DOI:** 10.5256/f1000research.183376.r398785

**Published:** 2025-08-23

**Authors:** M Mukhibat Syaufa

**Affiliations:** 1 Institut Agama Islam Negeri (IAIN) Ponorogo, Ponorogo, Indonesia

The title needs to emphasize "mixed methods." The article's originality is limited due to its conventional theoretical framework. Some literature is appropriate but repetitive. The methodology needs clarification and data integration. The results are interesting, but the discussion lacks depth. The scientific implications need further development. The references are not up-to-date. Analysis and consistency need to be improved

Is the work clearly and accurately presented and does it cite the current literature?

No

If applicable, is the statistical analysis and its interpretation appropriate?

Yes

Are all the source data underlying the results available to ensure full reproducibility?

Yes

Is the study design appropriate and is the work technically sound?

Yes

Are the conclusions drawn adequately supported by the results?

Yes

Are sufficient details of methods and analysis provided to allow replication by others?

Yes

Reviewer Expertise:

My research focus is on education, Islamic education management, education evaluation, teacher education

I confirm that I have read this submission and believe that I have an appropriate level of expertise to confirm that it is of an acceptable scientific standard, however I have significant reservations, as outlined above.

# Response

**Published:** 2025-08-25

**Authors:** Turyamureeba silaji

**Affiliations:** Foundations, Kampala International University - Western Campus, Bushenyi, Western Region, Uganda

1.We thank the reviewer for the insightful suggestion. The title has been revised to better reflect the methodological approach by explicitly including “mixed methods.”

**New Title:** *Evaluating the Types of Performance Monitoring Used in Private Chartered Universities in Uganda: A Mixed Methods Approach*

*2. *We acknowledge the reviewer’s concern. While Fayol’s and Vroom’s theories are well-established, we justified their use due to their relevance in assessing performance control and motivation. To improve originality, we have added commentary on the integration of these theories and their application in the Ugandan context.

 3. We appreciate this observation. The literature review section has been revised to eliminate redundant references and consolidate overlapping studies for better clarity and focus.

 4. We have revised the methodology section to clearly describe participant selection, data collection timelines, and how qualitative and quantitative data were integrated in both analysis and interpretation phases.

 5. Thank you for this valuable comment. We have expanded the discussion section to more critically interpret findings and relate them to existing literature, highlighting consistencies, contradictions, and contextual implications.

 6. We have added a section on the theoretical and practical implications of the findings, particularly how they inform the design of context-sensitive performance monitoring frameworks in developing higher education systems.

 7. Thank you for this important suggestion. We have reviewed and updated the references to include relevant studies published within the last 3–5 years, especially on performance monitoring and mixed methods research.

 8. We appreciate the reviewer’s feedback. We have carefully reviewed the analysis section to ensure consistent interpretation of both qualitative and quantitative findings, and strengthened the coherence across the results and discussion sections.

 dear reviewer, we appreciate and are grateful for the guidance given. my regards

# Response

**Published:** 2025-09-28

**Authors:** Turyamureeba silaji

**Affiliations:** Foundations, Kampala International University - Western Campus, Bushenyi, Western Region, Uganda

**Response to Reviewer Comments**

 Dear Reviewer,

 We sincerely appreciate the time and effort you have dedicated to reviewing our manuscript titled *“Evaluating the Types of Performance Monitoring Used in Private Chartered Universities in Uganda: A Mixed-Methods Study.”* Your insightful feedback has been invaluable in improving the quality and clarity of our work. Below, we provide detailed responses to each of your comments:

1. **Title needs to emphasize “mixed methods”:**
  We have revised the title to explicitly highlight the mixed-methods approach:
**New Title:** *“Evaluating the Types of Performance Monitoring Used in Private Chartered Universities in Uganda: A Mixed-Methods Study.”*
2. **Article’s originality is limited due to conventional theoretical framework:**
  In response, we have expanded the theoretical framework to integrate **Contingency, Agency, and Expectancy theories**, explicitly linking them to the mixed-methods design and the empirical findings. This enhances the originality by demonstrating a nuanced approach to understanding performance monitoring in private universities.
3. **Some literature is appropriate but repetitive:**
  We reviewed the literature and removed repeated citations while including **recent studies (2020–2025)** relevant to performance monitoring, academic staff performance, and ICT/e-learning systems. This ensures the literature review is both current and non-redundant.
4. **Methodology needs clarification and data integration:**
  We clarified our **mixed-methods approach**, detailing how quantitative (descriptive statistics, ANOVA, regression) and qualitative (thematic analysis) data were collected, analyzed, and integrated. The sampling strategy, data sources, and analytical procedures are now explicitly described to ensure methodological transparency.
5. **Results are interesting, but discussion lacks depth:**
  We have expanded the discussion section to provide **in-depth interpretation of findings**, comparing results with both regional and international studies, and highlighting implications for theory and practice. Each key finding is now thoroughly contextualized and linked to the literature.
6. **Scientific implications need further development:**
  The manuscript now includes a dedicated section on **scientific and practical implications**, emphasizing how the study contributes to performance monitoring theory, informs policy for private universities, and guides managerial practices in higher education.
7. **References are not up-to-date:**
  All outdated references have been updated or replaced with **recent peer-reviewed studies from 2020–2025**, ensuring that the manuscript reflects the current state of research.
8. **Analysis and consistency need to be improved:**
  We enhanced **analysis clarity and consistency** by standardizing terminology, aligning results with tables and figures, and ensuring a coherent narrative across quantitative and qualitative findings. This strengthens the rigor and readability of the manuscript.
9. **Overall presentation and citation of current literature:**
  The manuscript has been thoroughly revised to ensure it is **clearly and accurately presented**, with all claims supported by up-to-date literature.

We believe these revisions address the reviewer’s concerns and substantially improve the quality, clarity, and originality of the manuscript. We sincerely thank you again for your valuable feedback and constructive suggestions.

 Sincerely,

 Turyamureeba Silaji

silaji.turyamureeba@studwc.kiu.ac.ug

silaji.turyamureeba@studwc.kiu.ac.ug: Corresponding Author.

silaji.turyamureeba@studwc.kiu.ac.ug: Kampala Internal University.
