# Supplementary material for: Evaluating the Types of Performance Monitoring Used in Private Chartered Universities in Uganda: A mixed-methods
Source: F1000Res. 2025 Jul 8;14:675. [Version 1] doi: 10.12688/f1000research.166395.1 (PMC12658881; doi:10.12688/f1000research.166395.1)
Supplement: Peer review history

# Response

**Published:** 2025-09-28

**Authors:** Turyamureeba silaji

**Affiliations:** Foundations, Kampala International University - Western Campus, Bushenyi, Western Region, Uganda

**Response to Reviewer Comments**

 Dear Reviewer,

 We sincerely appreciate the time and effort you have dedicated to reviewing our manuscript titled *“Evaluating the Types of Performance Monitoring Used in Private Chartered Universities in Uganda: A Mixed-Methods Study.”* Your insightful feedback has been invaluable in improving the quality and clarity of our work. Below, we provide detailed responses to each of your comments:

1. **Title needs to emphasize “mixed methods”:**
  We have revised the title to explicitly highlight the mixed-methods approach:
**New Title:** *“Evaluating the Types of Performance Monitoring Used in Private Chartered Universities in Uganda: A Mixed-Methods Study.”*
2. **Article’s originality is limited due to conventional theoretical framework:**
  In response, we have expanded the theoretical framework to integrate **Contingency, Agency, and Expectancy theories**, explicitly linking them to the mixed-methods design and the empirical findings. This enhances the originality by demonstrating a nuanced approach to understanding performance monitoring in private universities.
3. **Some literature is appropriate but repetitive:**
  We reviewed the literature and removed repeated citations while including **recent studies (2020–2025)** relevant to performance monitoring, academic staff performance, and ICT/e-learning systems. This ensures the literature review is both current and non-redundant.
4. **Methodology needs clarification and data integration:**
  We clarified our **mixed-methods approach**, detailing how quantitative (descriptive statistics, ANOVA, regression) and qualitative (thematic analysis) data were collected, analyzed, and integrated. The sampling strategy, data sources, and analytical procedures are now explicitly described to ensure methodological transparency.
5. **Results are interesting, but discussion lacks depth:**
  We have expanded the discussion section to provide **in-depth interpretation of findings**, comparing results with both regional and international studies, and highlighting implications for theory and practice. Each key finding is now thoroughly contextualized and linked to the literature.
6. **Scientific implications need further development:**
  The manuscript now includes a dedicated section on **scientific and practical implications**, emphasizing how the study contributes to performance monitoring theory, informs policy for private universities, and guides managerial practices in higher education.
7. **References are not up-to-date:**
  All outdated references have been updated or replaced with **recent peer-reviewed studies from 2020–2025**, ensuring that the manuscript reflects the current state of research.
8. **Analysis and consistency need to be improved:**
  We enhanced **analysis clarity and consistency** by standardizing terminology, aligning results with tables and figures, and ensuring a coherent narrative across quantitative and qualitative findings. This strengthens the rigor and readability of the manuscript.
9. **Overall presentation and citation of current literature:**
  The manuscript has been thoroughly revised to ensure it is **clearly and accurately presented**, with all claims supported by up-to-date literature.

We believe these revisions address the reviewer’s concerns and substantially improve the quality, clarity, and originality of the manuscript. We sincerely thank you again for your valuable feedback and constructive suggestions.

 Sincerely,

 Turyamureeba Silaji

silaji.turyamureeba@studwc.kiu.ac.ug

silaji.turyamureeba@studwc.kiu.ac.ug

silaji.turyamureeba@studwc.kiu.ac.ug
